# The Hospital. Nursing Section

**Published:** 1902-10-25

**Authors:** 


### The Hospital.

### Nursing Section.

Contributions for this Section of "THE HOSPITAL" should be addressed to the EDITOR, "THE HOSPITAL" NURSING SECTION, 28 & 29 Southampton Street, Strand, London, W.C.

No. 839.—VOL. XXXIII. *SATURDAY, OCTOBER* 25, 1902.

### Notes on News from the Nursing World.

### THE MEMBERS OF THE NEW MILITARY NURSING SERVICE.

WE understand that Queen Alexandra's Imperial Military Nursing Service will, at the outset, consist of 27 matrons, 50 sisters, 200 staff nurses, and such first-class orderlies and non-commissioned officers as are selected. This number will probably be considerably increased. Candidates for admission to the service should apply for forms of application, with rules and regulations, to the Matron-in-Chief, Horse Guards, Whitehall, S.W.

### PRINCESS HENRY OF BATTENBERG AND WEYMOUTH NURSES.

ON Monday, when Princess Henry of Battenberg visited Weymouth to unveil the statue of the late Queen Victoria, the nurses of the Weymouth Trained Nurses' Institute were favoured with permission to present to Her Royal Highness a bouquet. The bouquet, which was composed of carnations, lilies-of-the-valley, roses and ferns, and tied with broad ribbons of green and pink, was presented by the matron (Nurse Cornwall) on behalf of the superintendent and nurses. When presenting the bouquet Nurse Cornwall said: "On behalf of the superintendent and nurses of the Weymouth Trained Nurses' Institute, may I beg your Royal Highness to accept this bouquet, with our grateful thanks for coming here to-day." The Princess smiled most graciously, and replied: "I am very pleased; thank you very much."

### THE WOMEN'S MEMORIAL TO QUEEN VICTORIA.

THE handsome sum of £5,858 has been handed, on behalf of the Irish Committee of the Women's Memorial to Queen Victoria, to the Marchioness of Londonderry, who is president of the central committee. As previously arranged, this amount will be invested by the trustees of the Jubilee Institute for Nurses, and the interest will be devoted to the work of the Queen's Home in Ireland. Meetings on behalf of the Memorial Fund were held at Hereford Ipswich and Norwich last week. On each occasion resolutions of sympathy were carried, and promises of support were tendered. The Norwich people are somewhat badly in want of increased funds for their own excellent hospital, but they are wise enough to recognise the fact that the nation, as well as the city, has claims upon their purses.

### A MAFEKING HEROINE.

IT will be recollected that the late Queen Victoria awarded the Royal Red Cross to Mother Teresa of the Sisters of Mercy at Mafeking, in recognition of the noble work which she did as the head of a little band of ladies, who, during the siege of Mafeking, nursed and cared for the wounded soldiers in the field. There has been unavoidable delay in making the formal bestowal of the decoration, and the ceremony was only performed last month by Lieut.-Colonel Vyvyan at the Convent in Mafeking. In the course of a brief address, Colonel Vyvyan pointed out that the motto on the cross is "Faith, Hope, Charity," and that these are also the three words forming the motto under which Mother Teresa and the sisters of the convent work and act. The Mayor of Mafeking and most of the prominent people of the town assembled at the convent on the occasion.

### THE NURSES OF THE IMPERIAL YEOMANRY HOSPITALS.

LADY HOWE, chairman of the Imperial Yeomanry Hospitals Fund Committee, requests that members of the staffs of the Imperial Yeomanry Hospitals who served in South Africa between January 1, 1901, and May 31, 1902, will send their present addresses and the dates of the commencement and termination of their services in these hospitals to Mr. Oliver Williams, 116 Victoria Street, Westminster, S.W., in order that the particulars may be compared with the entries on the medal rolls now being prepared by the commanding officers of the hospitals, in connection with the issue of the King's medal and clasps under the special Army Order of September 29, 1902.

### NURSING IN EGYPT.

IT appears that the supply of nurses in Egypt exceeds the demand. A correspondent in Alexandria who has nursed in Egypt summer and winter for some years, says she would not advise anyone to come out without having definite work in view. Each year the competition becomes keener, and it was pitiable last winter to hear of nurses who were thankful to take any kind of employment, such as needlework, in order to pay for the necessaries of life. A contributor to our columns recently spoke of nurses getting from three to seven guineas a week. The fees in Egypt, our Alexandria correspondent says, vary from two to four guineas; seven-guinea fees have been known, but only in exceptional cases, where nurses have gone very long distances and where there has been a heavy fare to pay out of it. She adds that "statistics show that a slack time for doctors and nurses generally follows an epidemic such as this country is now experiencing. There is every prospect, too, of the Cairo season being a late, and therefore a short, one."

### NURSING IN IRISH WORKHOUSE INFIRMARIES.

Last week we published passages from the report of the Irish Local Government Board which show that they are very much alive to the importance of improving the conditions of nursing in workhouse infirmaries as soon as possible. The suggestion that a conference should be held between the various teaching and administrative bodies concerned in the United Kingdom, with the view of arranging substantial uniformity as regards instruction and qualifications, coming from such a source, can hardly fail to be adopted. We note that in another part of their report the Irish Local Government Board says that, "while it is being realised that nurses for the sick, hardly less than physicians and surgeons, require to be systematically trained in their duties, many of the guardians still continue to resist the efforts to induce them to procure the appointment of qualified nurses rather than of pauper inmates, or of persons who, in many cases, are altogether unacquainted with nursing duties." This, of course, applies to England also; but such a state of things must soon come to an end, if the practice of the Irish Local Government Board—adopted since the passing of the Act of 1898—to refuse sanction to the appointment of any person, permanently or temporarily, to discharge nursing duties until it has been ascertained that she possesses the qualifications of a medical and surgical nurse, is in every case insisted upon.

### LITERATURE FOR NURSES.

The importance of a nurse adding to her scientific training a general culture cannot be doubted, and a carefully-organised effort in this direction which has been initiated on the other side of the Atlantic, merits notice on this side. At the Evansville Sanatorium in Evansville, Indiana, Dr. Edwin Walker has started a class in literature for the nurses who are undergoing training. It meets from eight to ten on the evenings of the second and fourth Tuesdays of the month, under the auspices of a director who arranges the programme, gives each of the members her share of special work, supplies the study books, and selects the supplementary reading. She also delivers the lectures which constitute the basis of the work, the superintendent of the nurses being always present. After each lecture there is a general discussion, each member of the class being required to express her views as a result of study during the interval since the last meeting. The discussion is followed by the reading of an item of current literary news by a member previously appointed. Then comes what is called "a story-telling drill." A previously appointed member tells to the rest of the class a story which she has read from one of the best magazines. It is usually of a humorous or dramatic character. To this succeeds a guessing contest, "which is intended to be both instructive and amusing." The members have to supply words omitted from quotations from books of the author under study. A "social half hour" closes the class. The first year's course is limited to American literature, the second to English literature, and the third to the world's great classics; and it is found that the introduction of the lighter elements at the meetings increases, rather than detracts from, the interest in the more serious part of the evening's programme.

### DUDLEY GUARDIANS AND THE SUPERINTENDENT NURSE.

In February last we published a long summary of the evidence given before the representative of the Local Government Board in support of certain charges made by the Dudley Board of Guardians against Miss Newbury, the superintendent nurse at the workhouse infirmary, and also the evidence in support of Miss Newbury's counter charges against the officials of the Dudley Workhouse. The result of the inquiry, as our readers will remember, was the vindication of the superintendent nurse. The Guardians had suspended her from her duties, but the Local Government Board instructed her to resume them, and found that while she had done nothing to merit suspension, she had on the other hand, proved that the administration of the workhouse was, in many respects, extremely lax. Of course Miss Newbury was re-instated because the Guardians could not help themselves, and apparently they have ever since been trying to get her to resign. At their last meeting they carried a resolution appointing the chairman of the Board, the chairman of the visiting committee, and the clerk, as a deputation to wait upon the President of the Local Government Board with a plea for her removal. Mrs. Powell, one of the guardians who is friendly to Miss Newbury, advises her in her own interests to resign, as in Mrs. Powell's opinion, she "will never have peace so long as she continues in the office;" but if she elects to stay we do not think that the Local Government Board, with their knowledge of the position, will permit Miss Newbury to be driven from her post.

### THE NURSES OF THE ROYAL HANTS COUNTY HOSPITAL.

As usual, St. Luke's Day was celebrated at the Royal Hants County Hospital as the anniversary of the foundation in 1736. The wards were gay with flowers and palms, and a festal spirit prevailed throughout. In the afternoon a service took place in the chapel, which was beautifully decorated. The nurses, who had been rehearsing for some time, formed the choir, under the direction of Canon Thompson, the former chaplain, the order of procession being probationers, staff nurses, sisters, matron, the hospital chaplain, the Bishop of Winchester and his chaplain. The singing of the hymns "Saviour, Blessed Saviour," "Ye Holy Angels Bright," "O God, our Help in Ages Past," and "Praise the Lord, ye Heavens Adore Him," was accompanied by violin and 'cello. An address was given by the Bishop on the "mending" of humanity and social conditions. A large number of friends and subscribers were present.

### LADY JEUNE AND THE QUEEN'S NURSE.

According to Lady Jeune, the Queen's nurse who works in her parish not only nursed over a hundred cases of measles with complete success during a recent epidemic, but continues, by her tact, to unite Church people and Nonconformists in a remarkable manner. Lady Jeune says, "There are few parts of England where Dissent was more virile and rampant than in our parish, and we watched with increasing interest to see whether the nurse's work would soften the friction which existed between church and chapel. The Dissenters now like her as much as Church people, and by the action of the leading Dissenter, who voluntary had a collection in their chapel on the same Sunday as the Church collection, a practical proof was afforded that, however wide might be the divergence of opinion on other subjects, there was none as regards the nurse."

### THE SUPERINTENDENT OF PLYMOUTH WORKHOUSE INFIRMARY.

The Hospital Committee who have been engaged in investigating the causes for the recent resignations of nurses at Plymouth Workhouse have reported that they are satisfied with the reasons given, and have expressed confidence in the management of the superintendent nurse. Judging from Miss Holliday's letter to the Guardians, she has had far from an enviable time during the last 19 months. Many changes have had to be made in the interests of the Board, the patients, and others concerned with the working and management of the infirmary. Miss Holliday says: "I have studied both patients' and nurses' comforts in every way possible. I have had no complaints, friction, or unpleasantness." Mr. Argall expressed confidence in the superintendent nurse, but differed from her in the manner she ruled. He denied that everything in the hospital was satisfactory, and declared that rules had been laid down which it was difficult for any nurse to work under. Mr. Hawke said that the statements regarding the management of the hospital were unfounded, and that the nurses who had resigned wanted a change. In the alterations she had made, Miss Holliday had endeavoured to give satisfaction to them and to the nurses. Dr. Lindsay was even stronger in his defence of the superintendent nurse, attributing the friction, or a large amount of it, to imagination. The Guardians have adopted the report, and a copy of the committee's resolution has been sent to Miss Holliday, whose letter is to receive the further consideration of the hospital committee.

### TRAINED NURSES FOR WIRRAL WORKHOUSE INFIRMARY.

It has been decided by the Wirral Board of Guardians to substitute for the present untrained nurses at the workhouse infirmary a trained superintendent nurse with two probationers to assist her. This is largely due to the fact that Dr. Yeomans, the medical officer, has tried for two years to work the place with untrained nurses and declares that he has found it is impossible to do so satisfactorily. The committee who had the matter under consideration were unanimous in recommending the change, and it is one that, in the interests of trained nurses, merits commendation up to a certain point. But the requirements of the case can only be fully met by the appointment of as many fully-trained nurses as the needs of the patients demand, and we are not at all inclined to favour the multiplication of training schools in small institutions. In fact, we do not think that the superintendent nurse at Wirral ought to be allowed, in the circumstances, to undertake the training of probationers.

### DISTRICT NURSING AT WARRINGTON.

At the fifth annual meeting of the Warrington District Nursing Association the report of the superintendent, Miss Whitfield, was read, and also that of the honorary treasurer. The former showed that 454 new cases had been nursed, and that between nineteen and twenty thousand visits had been paid. As far as the financial position is concerned there has been a gratifying growth of the subscription list, amounting to £66 10s. 6d., compared with that of the previous year. Since the accounts were made up the treasurer has received promises of £655 towards the purchase of the present nursing home or the building of a new one. From these particulars it will be gathered that the speakers at the meeting had some cause for congratulation. But the chairman was also able to announce that the Cycle Parade Committee had sent a donation of £75, a proportion of the proceeds of the recent carnival, and that further contributions to the Nursing Home Fund had been received. The rector of Warrington said that he could not express too strongly his admiration of the nurses. "Wherever he went he heard the people speak in great praise of their sympathy, tenderness, and kindness." In view of the handsome testimony which was borne on all sides to the value of the work, we hope that the "one large religious body in the town" which according to the chairman "has not yet contributed one half-penny to the institution from the beginning up to now" will see the error of its ways and speedily join with all the other religious bodies in supporting the association.

### KIDDERMINSTER INFIRMARY.

The foundation stone of the William Adcom Memorial Ward of the Kidderminster Infirmary has just been laid by the Mayor. In February, 1901, the town having decided to carry out some much-needed improvement to the infirmary as a fitting memorial to Queen Victoria, and the amount collected not being sufficient to defray the cost of the entire scheme, Mrs. Adcom, of Elderslie, generously offered to build and furnish a ward of six beds in memory of her late husband, the foundation stone of which was laid by his son, Mr. Peter Adcom. The new aseptic operating theatre is to be built over the William Adcom ward. The comfort of the nurses has also been considered and they are to have a new sitting-room and more bedrooms.

### SHORT ITEMS.

The following nurses arrived from South Africa on the *German*, which reached Southampton last Sunday:—Sisters H. T. Young, G. P. Gash, J. Cameron, and E. R. M. Bavan (Civil).—The Norwich Nurses' Co-operation has a new registrar in the person of Miss Edith Stuart Taylor, and the bureau is now established at No. 6 St. Stephen's Street. The hon. secretary is Miss Mottram, The Birches, Bracondale, Norwich.—The usual series of entertainments, organised for the amusement of the in-patients of the Cancer Hospital, Fulham Road, S.W., was successfully inaugurated on Tuesday evening last week, when "Liberty Hall" was presented by Miss Ethel Briscoe and the members of the Croydon Histrionic Society.—Over £18 was realised at the jumble sale held at Carnforth Lodge last week in aid of the Hammersmith and Fulham District Nursing Association.

### Lectures to Nurses on Medical Nursing.

By Eveline A. Cargill, M.D.Brux., L.R.C.P.&S.Ed.

### LECTURE IX.—DISEASES OF THE KIDNEYS.

The kidneys are the great excretory organs of the body, their function being to get rid of waste products. They are mainly concerned with the elimination of urea, a substance formed from the nitrogenous foods required for carrying on the work of the body. Urea and other waste products are thrown out by the kidneys in the urine, which is stored in the bladder until a sufficient quantity has accumulated, when it is finally evacuated through the urethra. This occurs about every four to six hours in health. The kidneys are two in number, situated deeply in the loin on either side of the spine. If one kidney is removed, or is so diseased that it cannot work, the other will enlarge and perform double work; but if both are diseased waste products accumulate in the system, and death results from uræmic poisoning.

Urine is secreted continuously, and passes slowly into the bladder, the secretion being involuntary until it reaches the bladder, but the evacuation from thence is under the control of the will. Patients may lose control over the bladder from various causes, the commonest cause being over-distension. When from any reason the bladder cannot be emptied for a long time it becomes so stretched that it is paralysed, and loses its power of contraction, and ever after urine is apt to dribble away. In cases of advanced paralysis, or injury to the lower part of the spine, there is loss of control over both bladder and rectum; urine and fæces are passed involuntarily or unconsciously by the patient. The following conditions are important for a nurse to remember:—When a patient does not pass water for many hours, and the fact being reported to the doctor, a catheter is passed; if urine flows it shows there has been *retention* of urine, but if no urine flows, and the bladder is empty, a much more serious condition is present—*i.e.*, *suppression* of urine. In retention the bladder only is at fault; it often occurs after operations, and is mainly a nervous condition; but suppression means that no urine is being secreted by the kidneys, therefore poisonous matters are being circulated in the blood, and the patient's condition is one of extreme gravity. *Incontinence* of urine means that urine is passed involuntarily, and it may dribble away continually. It occurs either with a very small bladder or one which is unable to retain any urine, or else the bladder may be distended and over-full, and the surplus urine runs away. The last condition is that of *retention with incontinence*.

A nurse should know the characters of healthy urine, and how to examine an ordinary specimen. Urine in matter is a clear yellow or straw-coloured fluid, having a specific gravity (as tested with the urinometer) of 1015 to 1025. It should be faintly acid, that is to say, that when a piece of blue litmus paper is dipped into it, it should turn red, and there should be no change on heating or cooling. The average quantity passed in 24 hours is 50 ounces, of which water forms the greater part, with a small proportion of solids, the most important being urea and salts. Urea is a nitrogenous compound, and is the form in which nitrogen is excreted from the body. It is derived from the proteids of the food, which are assimilated by the tissues; then the nitrogen reaches the liver, and is there formed into urea, which is carried by the blood to the kidneys, to be there separated from the blood, and finally eliminated from the body. The quantity so eliminated is about 500 grains a day.

All these characters may vary, even in health, much more markedly in disease. The quantity of urine may be enormously increased in hysteria, in diabetes, and in some forms of Bright's disease, or decreased in fevers and acute nephritis. The colour may be greenish (bile), smoky or red (blood). The specific gravity if very low points to Bright's disease, if over 1030 to the presence of sugar (diabetes). There may be a deposit on standing; in gouty persons of sedentary habits a copious white or pink deposit occurs as the urine cools and dissolves again on heating (urates); uric acid in excess is deposited in scanty red grains; phosphates form a thick white cloud in alkaline urine. Some substances found in the urine point to actual disease; *blood*, if hæmorrhage occurs in any part of the urinary tract blood appears in the urine; a small quantity makes "smoky" urine, a larger quantity gives it a dark porter colour, and blood corpuscles can be seen under the microscope. A clear deep red urine accompanies the disease called "hæmoglobinuria," while from the bladder or urethra blood may appear in clots.

Pus from any part of the urinary tract is deposited as a thick white sediment showing pus cells under the microscope.

*Albumen.*—When the kidneys are not acting properly, part of the proteids of the blood are allowed to pass out into the urine. This albumen appears as a cloud on boiling the urine, and always denotes disease.

*Sugar.*—If urine containing sugar is boiled with Fehling solution a precipitate of reddish brown or yellow grains is seen due to the action of copper in the solution on the sugar in the urine.

In putting up a specimen of urine for testing a nurse should see that it is passed into a perfectly clean vessel, the glass or bottle to contain it should be cleansed by the nurse herself, and the specimen conveyed to the doctor as soon as possible, as sometimes important changes take place in a few hours. The urine passed before breakfast and after breakfast mixed together is the best for testing. If the total quantity passed has to be measured it should be collected in a glass vessel and protected from dust, etc., and kept in a cool place.

*Diabetes* is not a disease of the kidneys, but of the liver; but as the most prominent symptom is the passing of a very large quantity of urine containing sugar, it is convenient to describe it in this place. In diabetes the "glycogenic function" of the liver is disturbed, and instead of sugar being given out to the tissues in small quantities as required, it is thrown out in such large quantities that it cannot be used and is carried away and thrown off by the kidneys in the urine. As a very large proportion of the total diet is diverted from its proper uses in this way, the patient soon becomes emaciated, and only very strict dieting will prevent him from wasting away. No starch or sugar can be allowed as these substances only feed the disease; so bread, flour, potatoes, fruit are forbidden. The nurse cannot prescribe diabetic diet, but she should know the principles on which it is formed so as to act intelligently in enforcing it, and to encourage the patient to persevere. It requires a great deal of tact to get a patient to put up with it, as it is very monotonous and uninteresting. The great difficulty is to find a substitute for bread, almond flour is mostly used. Modern treatment is very exact; a careful calculation is made of the amount of sugar thrown off daily by the patient, and the diet is proportioned accordingly in exact quantities which have to be weighed out; so much meat and so much bread, toast, or whatever substitute is allowed. It is better for diabetic patients to eat alone, as they get very hungry, and the temptation to indulge in forbidden luxuries is almost irresistible. This is specially the case with children. Young patients are easily cured; in old people the disease can be more easily kept in abeyance.

### Resignation of the Lady Superintendent of Charing Cross Hospital.

BY A FORMER SISTER.

Miss H. A. C. Gordon has resigned the post of lady superintendent of Charing Cross Hospital. The announcement will be received with universal regret by all who have been associated with her.

During Miss Gordon's long term of office one of the most marked improvements has been the altered conditions of the nursing arrangements.

When she entered on her duties in 1889 she had to contend with many difficulties. The hospital, as most people will recollect, had been under the care of St. John's Sisterhood, and although all praise is due to the methods of these ladies, radical changes were imperative to render Charing Cross an "up to date" institution. In order to effect them the newly-appointed lady superintendent had to exercise the greatest tact in her administration, while at the same time the full force of her clever organisation was required to set things going in proper methods.

It is as an organiser that Miss H. Gordon has been pre-eminently successful; her quick perceptions and clear deductions have been of immense value in her position of authority, and to many of her nurses these characteristics have proved a perfect tower of strength, not only in relation to their professional duties, but also in matters concerning their private life.

As a superintendent of nurses she has been sympathetic and kind, while her sense of justice has been one of her great attractions in the eyes of her staff, affording as it did to her subordinates a guarantee that they had someone to appeal to who would listen to both sides of a question.

In the matter of the training of probationers, Miss Gordon's influence has been largely felt in various directions. Her nurses have not only been trained to be efficient, they have been taught to be refined and quiet in their manners, and dainty and modest in their methods. These attributes, combined with complete practical knowledge of nursing, are the features of Miss H. Gordon's training, and if her nurses are careful to carry out her precepts their success will be assured.

As regards recreation, Charing Cross nurses have been peculiarly favoured during Miss Gordon's superintendence. Days off duty have been plentiful, and extra holidays have been freely given. These benefits have ensured the good health and happiness of the nursing staff.

Unfortunately, during the past few months Miss Gordon has been unable to discharge her duties on account of severe illness, and it will be the sincere hope of all that the rest which she has so well earned will completely restore her, and that she may enjoy many years of good health in a sphere of greater leisure.

### The Nurses' Co-operation.

For the last two or three years there has been considerable friction between the nurses and the Committee of Management of the Nurses' Co-operation, culminating last year in what was practically a revolt of the nurses. Among the demands of the nurses the chief two were that the paid secretary should not be a voting member of the Co-operation, and that the nurses should have a larger representation on the Committee of Management. The Board of Trade decided the first point, and the committee had to concede the second, and a meeting of members was called for October 7, 1902, at 5.15. But the first two points on the agenda were for alterations of the articles in the direction of making it more difficult to secure members of the Co-operation; and this had never been demanded by the nurses, and was a most unbusinesslike position to take up, as was shown by the fact that 10 members did not turn up to form a quorum and the meeting had to be adjourned. There are only 26 members of the Co-operation, and the present committee has refused to elect more members even when the names of medical men and former nurses have been submitted to them in due form; and yet there is no proposal to alter the articles which declare that the number of members is not to exceed 1,000; and that 20 members must sign a requisition for an extraordinary general meeting.

Let the nurses consider this carefully, for it is evident that the committee while conceding the right of more representatives on the committee have determined to keep the larger and confirming power of the general meetings entirely in their own hands.

The meeting of October 7th stood adjourned till October 15th at 5.15, and again no quorum was present between that hour and 6 P.M. During that time, however, I took the opportunity of pointing out my strong objections to the unnecessary first two resolutions, in conversation across the table. Also, in conversation, the solicitor's representative stated that if a quorum was not present at 5.45 the meeting legally stood adjourned. Meanwhile cabs and telephones were trying to gather together a quorum. At 6 P.M. 10 members not being present, I left; but information has since been received that a meeting was held. Now the 15th Article reads:—"If within half an hour from the time appointed for the meeting a quorum is not present, the meeting, if convened upon the requisition of members, shall be dissolved; but in any other case it shall stand adjourned to the same day in the next week, at the same time and place; and if at such adjourned meeting a quorum is not present, it shall be adjourned *sine die.*"

Therefore a notice has been sent to the secretary, to the solicitor, and to the Board of Trade, that the legality of the meeting of October 15th will be challenged. There are two important points for the nurses to note:—(1) The Co-operation is now surrounded by similar institutions; it is burdened with a large and expensive home which, according to the last report, does not pay its way, and if the Board of Trade withdraw the licence, or if legal and other quarrels continue, the prestige of the Co-operation is bound to go down, and it is the nurses who will suffer in the end. (2) The little handful of members who are clinging so desperately to the control of affairs have not ceased the methods the nurses will well remember in connection with their attempts to secure a hearing on June 14th, 1901. Do the nurses mean to allow the control to remain in such hands?

In conclusion there is the much regretted resignation of Nurse Geddes to record—Nurse Geddes who so ably led the agitation of last year.

(Signed) Honnor Morten.

[The Nurses' Co-operation was founded in 1891, and incorporated in 1894. It is the largest association of trained nurses doing private nursing in the world. It was established by a few ladies and gentlemen interested in the welfare of private nurses for their sole benefit and protection. The income of the Co-operation approaches £50,000 per annum, and some eight thousand cases were nursed by its members in private families during the last 12 months.—Ed. The Hospital.]

### Words of Advice to Nurses.

BY ESTHER H. YOUNG.

LECTURE II.—UNWRITTEN RULES.

(*Continued from Page* 32)

In all concerning our daily life, whatever our occupation may be, a constant recollection of the presence of God will be found the chief aid to patience. We may be tempted to answer quickly, or to frown, or in some way to show impatience, but just one short ejaculatory prayer, just a thought of Christ, just one "upward glance" as it were, one entreaty for help, and the battle is won! We know that if we try this plan, it never fails, yet we do not always remember to make use of it.

(1) Courtesy towards patients.

Now, about Courtesy with reference to the manners of nurses towards their patients, has it ever struck you what a difference your manner makes? Your manner to your patient, be it of "the Borough" or the West End type, when he is ill and at your mercy, watching your every movement, may influence his whole life for good or ill. In hospital especially, you may do infinite good by being polite and courteous to your rough uncouth coster, or curly-headed factory girl.

When you take any meals to your patients, see that they have their locker cloths, etc., and all that they need. See that they can reach everything and are quite comfortable. This will take more time than putting the food down on their lockers, and going away—but it is the better way!

When your patients arrive, and are new and strange, receive them kindly, gently; find time to make some polite remarks and so set them at their ease; and their friends will go away feeling satisfied that they are leaving them in kind hands.

If a patient calls you off from some other work, check any hasty, off-hand remark, and do what you can to please him, even if it is not exactly what he wishes.

Don't always call your patients by their numbers (you would not like it yourself if you were ill!) but take the trouble to find out their names. To a new patient or to some one who is only partly conscious, a number will convey no meaning, whereas if you called them by name, they would very likely respond. Sometimes patients have been in hospital for months without the Head Nurse knowing their names.

Again, always speak kindly of your patients. Do not, if they are rude, repeat amusing stories about their rudeness, but rather show them by your manner how to improve, and then, forget that rudeness. Don't speak at meals of "That horrid No. 10!" Be extra polite to No. 10, and then, when he returns home, his wife and children will bless Nurse for the improvement she has wrought in that home life, and he will bless you, too!

Never look upon these people as cases for you to learn upon—they are "patients," or to give the real meaning of the word—"sufferers," men and women with human hearts and sensitive feelings, needing your help, and moreover, sympathy and tactfulness in your dealings with them. You come to hospital to learn nursing, and of course you obtain your knowledge by your patients, but at the same time (whether you realise it or not!) undoubtedly you are teaching them. They watch your every movement, and they hear all your remarks while you are doing "washings," (By the way, do you not dislike the expression: "We have so many washings?" It sounds as though the patients were to be treated like mackintoshes or locker-tops!)

Be very considerate for the feelings of your patients; don't hurry them more than is absolutely necessary, and never, when matron comes in to go round the ward, leave a bit of that "washing" uncovered, or the feet bare, or wet. Of course matron wants someone to take her round, but she would much rather wait a few minutes than that any rudeness should be shown to the patients. I am sure she does not like "No. 6's" shoulders to be left half-covered, or "No. 2's" half-dried feet to be left showing, so please be careful in this respect, towards your sick folk.

(2) Courtesy towards friends of patients.

Now, as regards Courtesy towards the relations and friends of your patients, I would again advise carefulness about the "little things," when they come to inquire about anyone, you should go forward to find out what they want. If you are a probationer, and cannot help them yourself, go to someone who can, and ask them to wait for a minute or two while you take some trouble to find the sister or head nurse, Perhaps she will come and speak to the relatives herself, but if she gives you a message deliver it accurately and politely. Show some sympathy by your manner and do not just say: "Oh, he's all right," when you know nothing about it; or "She is going on well," when you are quite ignorant of the condition of that particular patient.

When a patient's friends have to sit up all night make them as comfortable as possible, and do not let them think that they are a nuisance in the ward.

On visiting days be polite to the mothers and sisters and brothers; find chairs for them if you can; tell them the truth about their relations, but do so as kindly as possible; consider their feelings! If any fault has to be found, leave that to sister. Don't say, "He is so troublesome," or, "She is always screaming," to some anxious and fond mother.

(3) Then let us consider courtesy towards visitors to the wards.

Besides the friends and relations there are the other visitors in the wards, to whom all due courtesy should be shown.

Now, a few words about the matron's regular visiting. As we said before, she will probably like to have someone to take her round, and she will wish to be told about anything which concerns the patients—but in telling her anything which you may think to be of interest, mind that you speak in such a way that they do not overhear your remarks. If anyone is not doing well, don't say out loud: "I don't think he'll last much longer." But still, if sister is absent, try to tell matron intelligently all that you know about the patients she is visiting. If matron comes round at night, and it is your duty to go forward and bring your lamp, do remember to keep the light off the patient's face, and do not say, "He is very bad to-night," he may hear you!

When visitors from outside are being taken over the hospital, go forward when they enter the ward and do what you can to show courtesy. Sometimes it is a very small act of kindness or politeness that is necessary, perhaps it is merely to tell sister that visitors are there!

(4) Courtesy of nurses towards one another.

And now let us consider our attitude, tone, manner, towards one another in our daily life in hospital.

Remember, each one forms a part of a great whole, each one is a member of a grand body—that of nurses—each one is a member of a large family. So much depends on the character of each individual nurse; and here, as in other matters, whether you know it or not, you set an example, and others are influenced for good or ill by you. You can't help it; you can't get away from the fact; you live in public and cannot avoid others seeing you, often following your example and being influenced by your example.

### WORDS OF ADVICE TO NURSES—*Continued.*

As nurses we live together in a very busy, hurried life, full of constant interruptions—and it is so difficult, isn't it, not to be impatient? Nurses are so apt to think that because they are nurses it does not matter, and that if they conform to rules of politeness towards their superiors this is enough. But it is not enough! You must try always, at all times, to be courteous to one another—at meals and in your dormitories, whether you are many together or just a few. I mean, for instance, when some of you are discussing your companions and so-and-so is being "picked to pieces." If you cannot take her part or do not think well of her yourself, keep silence, decline to talk unkindly of her, and don't say, "Oh, I can't stand nurse So-and-so!"

In some hospitals, before you are passed to sign as nurses, you have to go to the medical officer, and one of the first things he does is to look at your tongue, and examine your mouth, and then you have to go to the dentist, to have your mouth passed as being surgically clean. I often used to think, as I went through these formalities with nurses, that they were very significant. Recall it to your mind sometimes, the memory of thoughts connected with the "clean tongue" may sometimes check unkind gossip.

Never "cut" anyone. Perhaps it is difficult, but you should always be on speaking terms with each other. Again, if you are busy when sister calls you, don't say: "Bother!" (no, nor even look it!) but go to her at once, and answer politely. I know it is very difficult to be always courteous, in the midst of constant interruptions and very varied duties, but we must endeavour to be polite to all who are brought into contact with us, even in times of great pressure.

Do you know the "Legend of the Golden Letters"? A lady was once engaged at her prayers, when a nobleman who had heard that she was a very disagreeable woman, called to see her. She went to him, and entertained him so well that he was charmed. On her return to her room, she found her prayers printed in letters of gold—a token of God's acceptance of her act of courtesy and self-denial. There are many versions of this legend. (I daresay you know Longfellow's poem, "The Legend Beautiful") but whatever the setting, the principal thought, the lesson is the same—and we shall do well to remember it on days when we are especially busy, and consequently more likely to fail in showing politeness.

In the home, ask the home sister for anything you may need, but ask politely. At your meals, don't toss your plate away, or grumble openly at the housekeeper, or send rude messages by the servants, but ask politely for what you want, and you will find that you also will be treated with politeness and consideration.

Avoid "talking shop" at meals. It is not quite polite, even before nurses, and if you get into the habit while you are in hospital, you will forget and do the same thing at other tables, when it will be considered very rude and ill-mannered on your part.

Those of you who have to teach and train juniors, should always correct others with as much courtesy as possible. New "pros." are "dreadful" of course, but you were one once, nurse, and probably you did some stupid things, too! And had nurse no patience with YOU? Be very patient and kind and gentle now, in your turn, and if you find your young probationers are a great worry, follow the advice of the old Chinese proverb: "Think of your own faults when you go to bed, and are awake, and cannot sleep, but think of the faults of others, later on, in the night, when you are asleep!"

### The Nurses of Northampton General Infirmary.

### INTERVIEW WITH THE LADY SUPERINTENDENT. BY OUR COMMISSIONER.

THE nursing at Northampton General Infirmary is necessarily under present conditions carried on at a serious disadvantage, and the completion of the new building will afford the much-desired opportunity of effecting many reforms. The information which I obtained from the superintendent of nurses on the occasion of my visit may, to some extent, help to indicate the directions in which improvements will be not only practicable but essential, as soon as Northampton is in possession of an infirmary worthy of one of the most enterprising towns in the kingdom. There is according to the existing rules of government a matron as well as a superintendent of nurses, and the present matron, Miss Pell, is a lady who has occupied the position for nearly forty years to the satisfaction of the governors, the staff, and the inmates. But her obligations are limited to domestic affairs and to the responsibility of seeing that meals are provided at the proper hours. The lady superintendent, Miss Marian Necpe, has absolute control over the nurses, probationers, and ward servants, with her separate rooms, and, generally speaking, the authority which ordinarily belongs to the matron of a general infirmary. When I had seen for myself some of the difficulties and drawbacks of the situation I inquired about the system of nursing.

"For ten years certificates have been given," replied the superintendent. "We made a start with two years' training, but the period has been increased to three. Nor have we stood still in respect to the number of nurses. Since I have been here the staff has been augmented by nine, and a night superintendent has been added."

"You have been here, I think, since the training school was established?"

#### THE ELECTION OF SUPERINTENDENT.

"Yes, I came in March 1892. I was trained at the London Hospital, and went from the London to Charing Cross, where I was sister for about four years. Subsequently, I was night superintendent at the Royal Infirmary, Wigan, an appointment I continued to fill until I was elected superintendent of nurses here. I think that the mode of electing the superintendent might be altered with advantage."

"Perhaps you would describe the mode?"

"The whole body of 75 governors have to be canvassed. My election was not contested, but if it had been, I should have had to call personally on the electors, some of whom reside at a great distance. Even in my case, the printing and stamping of proxy papers and other expenses amounted to about £12, which means a considerable slice off the modest salary of the superintendent."

#### THE SISTERS.

"Is the staff, as enlarged, adequate?"

"Now that the committee have granted nine sisters it is. At present we call them nurses, but when the new building is opened, they will be generally designated sisters. During the day each sister has charge of a floor, with thirty beds. But thirty beds in small wards with long passages to traverse, means much more than thirty beds in a big ward. There is a sister in charge of the children's ward with nineteen beds,

### THE NURSES OF NORTHAMPTON GENERAL INFIRMARY—*Continued.*

and another in charge of the accident ward with sixteen beds."

"And at night?"

"The night superintendent has charge of the whole building. In spite of having to sleep close to the ward, which often contains delirious patients, and adjoining the ward scullery, the present night superintendent has been here three years."

"Were the nurses who discharge sisters' duties trained here?"

"I prefer, if I can, to have sisters who have been trained here, and several old probationers have come back again. The entire number of nurses is thirty-five. Seven probationers are on duty at night, but as a rule, no probationer is allowed to do night work until she has been in the infirmary six months."

### Night and Day Duty.

"How long does a probationer remain on duty at night?"

"For three months, and there are lamentations when the period ends. Night duty seems to suit our nurses, and they certainly like it better than day duty. They go on duty at 9 P.M., after breakfasting at 8.30, and off at 9 A.M. They have one night off each month, and every morning they have from 10.30 to 12.30. Thus, the night off means that they can get away at 10.30 in the morning, and need not be back until 2 P.M. the next day. They appreciate this arrangement very much, as they can get right away to London and elsewhere by one of the many cheap trains."

"Is there any trouble about diet?"

"None at all. During the night they can have tea or cocoa, and they take up some food from the dining-room which they have in the wards, such as meat, eggs, and blanc mange. At 10 they have dinner. They are expected to be in bed at 1, and are called at 8 P.M."

"What are the hours of the day nurses?"

"They breakfast at 7, go on duty at 7.30, have lunch in the wards, dine in two detachments at 12.45, or 1.15, tea at 4 or 4.30, and supper at 9."

### The Meals.

"Who presides at the meals?"

"The sisters take it in turns to preside at tea for the entire time, and the sister who presides at tea carves at breakfast. The night sister presides at breakfast. When I came here the nurses had breakfast, tea, and supper in little rooms off the wards, with the result that anyone going round at 11 o'clock at night could see all the leavings from the meals on the table."

"I make a point" continued the superintendent "of the nurses being down to meals punctually, and of seeing that they eat their food when it is given to them. If they are wasteful in respect to their own food, they will be the same with regard to that of the patients. It is very requisite to teach them to be careful in little things here, for money is by no means easy to get. The food is plain but very good, and there is always plenty of it. The chairman and the house committee are most anxious that there should be no stint, and also that the conduct of the nurses should correspond in excellence with their work."

### Off Duty and Holidays.

"When are the day nurses off duty?"

"Generally speaking in the afternoon from two to four. They have a day off once a month, from 10 A.M. until 9.50 P.M.; and every other Sunday from 2 until 9.30. The sisters are off duty from 2 until 9.30 every other Sunday, every day from 5 until 7, and half a day once a week."

"Are the day nurses always obliged to be in at 9.30 P.M.?"

"It is necessary that they should be in about that time as the porter goes to bed at ten. Sometimes they have permission to visit a theatre, and then I like three or four to go at once, so that the porter may be disturbed as little as possible. As to holidays, the probationers have a fortnight in the year, and the sister three weeks. But they all have to take their holidays when the Infirmary is being cleaned, because at that time, between May and August, many of the wards are closed."

### Salaries and Uniforms.

"What about salaries?"

"The probationers receive £5 at the end of the first year, £14 the second year, and £18 the third. The sisters have £28 the first year, £30 the second, and further increases are made at the discretion of the Committee."

"Do you take paying probationers?"

"We very rarely take them, unless someone in the locality, who wants to engage in mission work, wishes to be trained for six months. Pupil nurses are required to provide their own uniform."

"Is it obligatory to wear uniform out of doors?"

"Yes, except when a nurse is cycling. Indoor uniform is provided by the Governors, consisting of 16 yards calico and three print dresses a year. The outdoor uniform, which the nurses have to provide for themselves, is a green dress, black cloak, bonnet and veil, with white strings.

### Training and Certificate.

"Do you get plenty of applications for probationership?"

"Sometimes there is a dearth, and at others we have more than we want. Before a probationer is engaged a number of questions, which I found here when I came, are asked about her character. The applications are not usually local."

"I conclude that as many of the nurses are away for their holiday between May and August, there are no lectures during that time?"

"No, we only have lectures in the winter months. I give them before Christmas, and the medical staff after. There is a written examination subsequent to each course of lectures."

"How does your certificate of training read?"

"Following a number and the name, it runs: 'This is to certify that —— has been trained as a nurse for three years in this Infirmary from —— to ——.' It is signed by the chairman of the House Committee, the physician and surgeon, and the superintendent of nurses. There used to be a space for remarks about work and conduct, but there were objections and we had to alter it."

### The Nurses' Quarters.

"After seeing the nurses' quarters, I need scarcely ask much about them."

"They are, of course, most inadequate. The probationers sleep in small cubicles curtained off, twelve in a compartment; there is only one bath-room for the whole staff, and the sisters' rooms are off the wards. The dining-room would be better if it had not also to be used for washing-up purposes."

"And the sitting-room?"

"Their only sitting-room is the room which was intended for convalescents. When they are away the nurses use it, and until three years ago they had nowhere to sit. The position is very inconvenient, owing to three of the night nurses having to sleep close to it."

"But," concluded the superintendent, "all these things, as well as the necessity of keeping articles of food in cupboards, will be rectified when the new infirmary is finished, part of the present one converted into a nurses' home, and we are provided with an isolation ward. We are all looking forward to the completion of the new building, which will not only be a great boon to the patients, but will also materially lighten the labours and add to the comforts of the nursing staff."

### Army Nursing, Past and Future.

BY A RESERVE SISTER.

Before criticising the new regulations for the Army Nursing Service, henceforth to be known by the somewhat unwieldy title of "Queen Alexandra's Imperial Military Nursing Service," it will be as well to consider the conditions obtaining under the old system and to compare the duties and responsibilities of sisters in civil and military hosbitals.

### The Former Duties of a Wardmaster.

An erroneous idea prevails that an army sister's duties were confined to the rôle of "the one who looked on." As a matter of fact an army sister could do even more for her patients than a sister in a civil hospital Thus, a great many of the duties for which, in a civil hospital, the sister-in-charge of a ward would be responsible, were discharged, in a military one, by the "wardmaster." The "wardmaster" was a non-commissioned officer—a sergeant or corporal as the case might be—and in small station hospitals was often the "compounder" or dispenser as well as wardmaster, and was also responsible for those wards not nursed by sisters. The wardmaster of a sister's wards was responsible for the cleanliness and order of the wards and their annexes, the discipline and behaviour of the patients, and the due performance by the orderlies of their duties. Any complaint the sister had to make with regard to the orderlies' work or patients' behaviour was made to the wardmaster. He was responsible for the proper supply of bedding and ward linen and had charge of the ward equipment. He had to see that each patient on admission was furnished with his hospital equipment of hospital clothes, bed-linen, and crockery, and had in return to receive from him his own clothes and any personal belongings he brought with him into hospital, giving him a receipt for the same and returning his own property to him on his discharge. It was the wardmaster's duty to see that the "diet summaries" were correctly compiled by the ward orderlies from the patients' diet sheets, and that the dispensary book went to the dispensary at the right time. He was also responsible for the due attendance of patients to be discharged at the office of the senior or principal medical officer at the proper hour.

### The Sister in a Civil Hospital.

In a civil hospital a great part of a sister's time is spent in attending the "honorary" or "visiting" physicians or surgeons during their clinical lectures to their students, this, of course, in addition to accompanying the resident physicians or surgeons in their morning and evening visits to their wards. In most military hospitals the principal medical officer, known as the "P.M.O.," who visits would correspond to those of the "visiting men," comes but once a week at the very most, and having no class of students to lecture to his visit is quickly over. Ward-books and diet-lists in a civil hospital give the sister much to do. In a military hospital the sister's book-keeping was *nil.* The admission and discharge books were kept in the registrar's office; the medical officer in charge of each ward kept his own case-books, and the wardmaster, as has been stated, was responsible for the diet summary and the linen. The "household" duties which a civil sister has to see to—the cleanliness, temperature, and ventilation of her wards, the eternal polishing of ward furniture and "brasses," etc.—were all part of the wardmaster's multifarious duties, not the sister's. Both civil and military sisters trained their nurses, whether probationers or orderlies, giving them lectures and practical instruction in their duties, bedmaking, washing helpless cases, bandaging, sponging, etc. But having the nursing only of her patients to attend to, an army sister could take charge of a far greater number of patients than would be possible in a civil hospital, and also could devote a great deal more of her time to the personal care of those sufficiently ill to require her to wash, feed, and sponge them, to make their beds and "do" their backs herself. She also took all necessary temperatures, pulses, and respirations, and administered all medicines and stimulants, and, in South Africa at any rate, did a very large proportion of the dressings. I have dwelt on the "sister's" duties alone because it is with regard to the sister's duties that the great changes provided by the new regulations will be made.

### The Matron-in-Chief and Matrons.

Let us now consider these rules. In place of the director-general and the lady superintendent at the head of the service there are a nursing board and a matron-in-chief. No one can have anything but praise for this arrangement. The matron-in-chief is without doubt the proper person to receive the "service records and confidential reports . . . on the sisters and staff nurses," and "to recommend the appointment, promotion, distribution, retirement and dismissal of members of the service" as provided by paragraphs 141 and 143, in addition to performing the other duties of inspecting hospitals, etc., which have always been carried out by the lady superintendent. The appointment of "matrons" appears to be only a change in name, as their duties seem to be identical with those of the "superintendent" or "acting superintendents" in the old service except that they are responsible for "the cleanliness and good order of the wards" under the various sisters' charge.

### The Principal Innovation.

The great change, with the exception of one to be dealt with later, lies in the fact that, by paragraphs 161, 162, b, c, d, e, f, g, h, i, j, k, 163a, 164, 164a and b, o, 165, 166, 166a, B, the sister is now responsible for all the duties previously discharged by the wardmaster, whose post is apparently abolished. Any complaints she may have to make are now to be laid before the matron, and not as, formerly, taken to the wardmaster or medical officer in charge of the ward. It is not necessary to recapitulate the wardmaster's duties: they are certainly those which every sister would desire to be responsible for. But it is no less certain that with the addition of all these new duties she cannot possibly give the same time either to the personal care of her patients or to the supervision of the orderlies' nursing, and the nursing board has decided to meet the difficulty by the innovation of staff nurses. On paper this sounds very reasonable, but in practice the difficulties of nurses and orderlies working together will probably be found insurmountable.

### Staff Nurses and Orderlies.

First of all these staff nurses are to be fully-trained women holding a three years' certificate from a recognised civil hospital, that is to say, they must possess the qualifications necessary for the important post of sister in a civil hospital. But they are required to enter on a further probationary period of two years in a military hospital before they have the chance of becoming sisters. These fully-trained nurses are to work, apparently, on an equality with the orderlies, sharing "the routine ward work." Their respective duties are not defined in the regulations for admission to the Q.A.I.M.N.S., though the orderlies may be in the "Standing Orders for the R.A.M.C. However, two

ARMY NURSING, PAST AND FUTURE—*Continued.*

most unfortunately-worded paragraphs Nos. 172B and 172C warn the staff nurse "to refrain from relegating an unfair share of the routine ward work to the orderlies" and "to take a full share in duties, which are necessary, however unpleasant." Surely no nurse worthy of the name needs to be told not to shirk unpleasant duties, nor to refrain from putting an unfair share of her work on other people, and one may hope that it is the first and last time that such an order will appear in the printed rules of any service or hospital. A man having completed three years' service in the R.A.M.C. has usually risen to the non-commissioned ranks, and would not be expected to work on a level with a private, and a nurse holding a three years' certificate is hardly likely to begin again as a probationer. In spite of her three years' training the staff nurse is not considered competent to measure poisonous drugs employed for hypodermic injections, sleeping draughts, etc., though probably in her civil hospital she has measured and given hypodermic injections and sleeping draughts. Paragraph 169 makes the sister personally responsible for the measuring of all poisonous drugs. The staff nurse is not considered competent to take charge of a case when an anæsthetic is given, though during her training she has very likely "taken" many important operations. Paragraph 171A provides that a sister must always be present, and if the ward sister is off duty and another sister not available "the staff nurse must give immediate notice to the matron that the necessary assistance may be supplied."

The Question of Uniform.

Staff nurses will be invaluable in charge of wards on night duty, for the orderlies alone are not sufficient even under the supervision of the night sister. The pay and allowances need no comment, but one minor detail as to dress may be noted. Paragraph 161A provides for the wearing of uniform on all occasions except when on leave. A sister is held to rank as a departmental officer of subaltern rank "but this regulation treats her as a private soldier." Beside this, wearing uniform in the streets makes a woman conspicuous, and the feeling of being an abject of notice is most unpleasant to any woman except one desirous of attracting attention to herself. It is to be hoped that the matrons will be given a wide discretion as to the enforcement of this rule.

### Everybody's Opinion.

**[Correspondence on all subjects is invited, but we cannot in any way be responsible for the opinions expressed by our correspondents. No communication can be entertained if the name and address of the correspondent are not given as a guarantee of good faith, but not necessarily for publication. All correspondents should write on one side of the paper only.]**

VILLAGE NURSES IN CUMBERLAND.

"Lady Knightley" writes from Fawsley, Daventry: I am sorry to see that your correspondents, whilst disagreeing about the advisability of employing fully-trained nurses for district work, agree that it is impossible for country districts to support them. Knowing how unfortunate it is for the cause of village nursing that such statements should appear in your paper, I venture to send an account of my own experience. Seven years ago a district nursing club was started on a provident basis in this neighbourhood. For the first year it consisted of one district, employing one nurse. Since then it has gradually increased, until now it consists of four districts, comprising 22 parishes, with a staff of four nurses. No nurse is, or has been, employed unless she has had three years' training in one of the best hospitals. The average cost of each nurse is about £80, and hitherto there has only once been a small balance on the wrong side at the end of the year, and that was owing to purely exceptional circumstances. I may add that the working people pay a subscription of 4d. a month for each family, farmers and tradespeople 10s. a year, and others not less than £1. These payments entitle the subscribers to the services of the nurse, whenever they like to send for her, without any further payment. I think the above facts are sufficient to prove that it is well within the bounds of possibility to maintain a fully-trained nurse in any rural district. This is by no means a rich neighbourhood, and most of the parishes are purely agricultural.

"R. D." writes: I have been working as district nurse in a scattered district in Cumberland for more than a year, and I think that the letter which appeared in a recent issue of The Hospital was intended to apply to me, and to the district where I was working. There has been a great deal of trouble in this district, and at present two nurses are working one against the other. One a Cumberland Nursing Association nurse; the second one working under a working-class committee. The work has been very successful, but it is quite true that several members of the local committee of the Cumberland Nursing Association have done their best to try to drive me away from the district. I may safely call myself a thoroughly experienced nurse. I have not the least idea who wrote the letter in the issue of October 4th, but it pointed so exactly to me and to my district that I feel bound to answer the letter in your issue of October 11th from Lady Lonsdale. I, however, do not wish to cast any reflection whatsoever on the nurses or committees of the Cumberland Nursing Association. The rules are very good, and I should have been very glad to work under their rules. I know they have very excellent nurses, but in this particular district feeling runs very strong on both sides. The nurses' life in consequence is very unpleasant. I have resigned and am leaving my district. If the letter was not intended for me and my district I must apologise.

THE FUTURE OF THE ASYLUM WORKER.

"Reform" writes: Seeing the able and sympathetic articles and letters in The Hospital the last few weeks on the better nursing of the insane, I beg as a nurse of six years' experience in the mental wards of a large Poor Law institution space for a few remarks. I certainly think that there is some room for improvement with regard to nursing mentally-sick cases. Nor is the administration faultless: the wards and surroundings in large town workhouses are generally dull and cheerless for patients suffering from nervous diseases. For the patients who can work the only employment is scrubbing, washing, and a little sewing; very little amusement is provided, and all kinds of cases are very much mixed up together. The nurses, where there is anything like a staff kept, are of a mixed class; they get no education nor relaxation except their off-duty time. The hours of duty are rather long, considering the nature of the work. If untrained men and women are engaged to nurse the patients they get no instruction except what they pick up from their fellow workers, though they receive the same salary as the nurse who comes with a psychological certificate. Mental work is interesting, and might be made more so. Many patients are sent to the workhouse in the first stage of insanity for observation, and often these cases are very ill physically. As "An Asylum Worker" points out some mental patients do not complain when sick, and on the other hand others fancy that they are ill; it is therefore necessary to be as observant as with children. I think that a mental worker should have a thorough knowledge of sick nursing; a double training is required. If a good part of the probation time could be spent in a large hospital, and the other part in the asylum wards, I believe that mental nursing would be improved.

### Appointments.

Allt-yr-yn Hospital, Newport.—Miss C. Terry has been appointed sister. She was trained for three years at the Royal Southern Hospital, Liverpool, and has since been charge nurse at the Northern Hospital, Winchmore Hill, N.; charge nurse at the Royal United Hospital, Ventnor; and private nurse at the Royal United Hospital, Bath.

Borough Hospital, Plymouth.—Miss Barrs has been appointed sister. She was trained at St. George's-in-the-East Parish Infirmary, London, and holds the bronze medal from that institution. She has since been nurse-matron at the

Brandon Cottage Hospital; charge nurse at the Eastern Hospital, Homerton; charge nurse at the Diphtheria Hospital, East Ham; holiday sister at the Royal Infirmary, Wigan; and she has also done some private nursing.

Indian Army Nursing Service.—Miss Mabel Stronghill has been appointed sister. She was trained at the Westminster Hospital, and for some time past was sister of the women's medical wards of the Queen Adelaide Institution.

Jessop Hospital for Women, Sheffield.—Miss Elizabeth Dale has been appointed staff nurse. She was trained at Chelmsford Infirmary, and has since been assistant nurse at St. Leonard's Hospital, Sudbury, and charge nurse at Chelmsford Infirmary.

Lloyd Hospital, Bridlington.—Miss Ada A. Hoghton has been appointed matron. She was trained for three years at the Victoria Hospital, Burnley, where she has since been sister of children's ward, sister of female ward with charge of operation theatre, and assistant matron.

Nottingham Children's Hospital.—Miss Francis has been appointed staff nurse. She was trained at St. Mary's Hospital for Children, Plaistow, for two years, and Derby Infirmary for six months.

Nurses' Co-operation, 8 New Cavendish Street, W.—Miss Bessie Chamberlain has been appointed senior assistant matron. She was trained at St. Thomas's Hospital, after which she did 15 months' district nursing in connection with the Queen Victoria's Jubilee Institute. She has since been junior assistant matron at the Nurses' Co-operation.

Paddington Infirmary.—Miss Lloydie Williams has been appointed night superintendent. She was trained at the Cardiff General Infirmary, and has since been charge nurse at the North-western Fever Hospital, and sister of the children's ward at Paddington Infirmary. She has also done private nursing.

West London Hospital.—Miss Theodora Unwin has been appointed night sister. She was trained at the Liverpool Royal Infirmary, and has since held the post of sister at the Royal Halifax Infirmary, and at the Cumberland Infirmary, Carlisle.

Worcestershire Sanatorium for the Open-air Treatment of Consumption, Knightswick.—Miss S. Balfour Hope has been appointed matron. She was trained at Glasgow Western Infirmary for four years, and has since been private nurse in connection with the Royal Scottish Nursing Institute, Edinburgh, matron and nurse superintendent respectively of sanatoria at Kingussie.

### Presentations.

Parish Infirmary, Portsmouth.—Sister Price, who is leaving the Parish Infirmary, to take up similar duties at the Infirmary, Shirley, has been presented with a writing-case from members of the nursing staff at Portsmouth.

Coathill Hospital, Coatbridge.—Miss Binnie, one of the staff of Coathill Hospital, Coatbridge, was last week the recipient of a handsome writing case on the occasion of her leaving to join the Sunderland Nursing Institution. Dr. Hamilton, the visiting physician of the hospital, in the name of the matron and nurses asked Nurse Binnie to accept the gift as a mark of esteem and goodwill and wished her every success in the post she was about to enter.

Durham County Hospital.—The much regretted departure of Miss Deakin, Matron of Durham County Hospital, was recently marked by a social gathering of the nurses and their friends to make a presentation to her in view of her approaching marriage to Dr. P. A. Pearcey, of Sunderland. From the house surgeon, sisters, and nurses she received a cut glass silver mounted vase, from the secretary and his wife a silver shell butter dish and knife, and from the domestic staff a beautiful silver gilt basket and sugar sifter.

### For Reading to the Sick.

"GRANT US THY PEACE."

Grant us Thy peace throughout our earthly life,
Our balm in sorrow, and our stay in strife;
Then, when Thy Voice shall bid our conflict cease,
Call us, O Lord, to Thine eternal peace.

*Hymns A. & M., No.* 31.

Peace He leaves us at His going; His peace He will give us when He comes at the end. Peace He leaves us in this world; His peace He will give us in the world to come. Peace He leaves us, in which, by abiding therein, we overcome the enemy; His peace He will give us when we shall reign as kings without an enemy. Peace He leaves us, that even here we may love another; His peace He will give us, where it shall never more be possible for us to disagree. Peace He leaves us, that we may not judge one another touching things which are secret to each of us, while we are in this world; His peace He will give us when He shall make manifest the thoughts of the heart, and then shall every man have praise of God. Yet in Him, and from Him, it is that we have peace, whether that which He leaves us at His going to the Father, or that which He will give us when we ourselves are brought by Him to the Father.

*St. Augustine.*

O wondrous peace, in thought to dwell
On excellence Divine;
To know that nought in man can tell
How fair Thy beauties shine!

O Thou, above all blessing blest,
O'er thanks exalted far,
Thy very greatness is a rest
To weaklings as we are;

For when we feel the praise of Thee
A task beyond our powers,
We say, "A perfect God is He,
And He is fully ours."

*Hymns A. & M., No.* 32.

When God is in the midst of a kingdom or city, He makes it firm as Mount Sion, that cannot be removed. When He is in the midst of a soul, though calamities throng about it on all hands, and roar like the billows of the sea, yet there is a constant calm within, such a peace as the world can neither give nor take away. What is it but want of lodging God in the soul, and that in His stead the world is in men's hearts, that makes them shake like leaves at every blast of danger?—*R. Leighton.*

O God! who gav'st Thy servant grace,
Amid the storms of life distrest,
To look on Thine Incarnate Face
And lean on Thy protecting Breast.

Be ours, O King of Mercy! still
To feel Thy presence from above,
And in Thy Word and in Thy Will
To hear Thy Voice and know Thy love.

*Heber.*

### Echoes from the Outside World.

### The Royal Drive through South London.

The King has appointed next Saturday, the day of the procession, to be a Bank Holiday throughout London. This order does not of necessity apply to any other towns in England, nor to the London suburbs. But it had been represented to the King that there would be a great element of danger were the banks to remain open along the line of route, and unless some definite order were given they could not be closed in some streets and open in others. Hence the proclamation. Those who have seats along the line of route are advised to be in their places by 9.45 A.M., but this does not, of course, apply to any which can be approached by back entrances. Queen Alexandra, accompanied by Princess Victoria, left Copenhagen on Monday morning by special train. All the members of the Danish Royal Family were present at the station to bid the Royal visitors farewell, King Christian being visibly affected at parting with his beloved daughter. The Queen reached Victoria Station on Tuesday evening, looking remarkably well, and was met by the King, and Prince Charles of Denmark. Their Majesties, preceded by a single mounted escort, drove in a closed carriage to Buckingham Palace.

### Movements of Royalty.

Princess Henry of Battenberg on Monday visited Weymouth and unveiled a statue of Queen Victoria which has been erected by public subscription as a Coronation memorial. It is of bronze, erected upon a pedestal of Portland stone, and the figure alone is over 8 feet in height, representing Her Majesty, standing erect, wearing the robes of State and Crown, and bearing the Sceptre and Imperial Orb. After the statue had been unveiled, Her Royal Highness afterwards expressed a wish to speak to any officer in attendance who had been to the war. Accordingly Captain Ingham and Colonel Symes were presented to her before she returned by special train to Southampton *en route* for Osborne. Both the Kaiser and the King of Portugal are expected in England next month, and their visits will be of a purely private character. The Kaiser is due on November 8th, and will go to Sandringham, after lunching at Shorncliffe with the officers of the 1st Royal Dragoons, of which regiment he is colonel-in-chief. He will remain in Norfolk for a week, will probably shoot daily, and then proceed to Lowther Castle, where he will spend a couple of days. King Carlos will probably arrive the day of the Kaiser's departure. He will go straight to Windsor, and remain there a week or ten days.

### Disaster in Somaliland.

News reached England on Monday that Colonel Swayne had met with a serious reverse in North-east Africa, two officers and 50 men being killed. As long ago as March 1899 the Mad Mullah or prophet named Haji Muhammad Abdullah, made his first appearance in Somaliland. He collected arms and men, and endeavoured to establish his power, temporal and religious, over the south-eastern portion of the Protectorate. He has been described as a man in the prime of life, tall and dark, and he began his operations with 3,000 men. In 1900 the English Government organised, in conjunction with the Abyssinians—Somaliland borders upon Abyssinia on one side—a small expedition against the fanatic and his followers. He fell back as the British approached, and little was done till the following year, when he was attacked by Colonel Swayne and defeated with heavy loss. As the Mullah himself was not captured, hostilities have continued, and now, according to an undated telegram, the British force, which consists entirely of black soldiers, officered by white men, is obliged to retire. Colonel Swayne has about 2,500 troops, of whom half are raw Somali recruits, who fight bravely, but want seasoned troops to support them. They have been apparently disheartened by the large force, 15,000 to 20,000, of the enemy, and Colonel Swayne does not dare to risk another engagement. Much hampered by the want of water and by the wounded, exceeding a hundred in number, the commanding officer is working his way to Bohotle. He asks for immediate reinforcements, which have since been sent from India to Aden. Considerable anxiety will be felt till tidings of the safety of the expedition are received, as the country is swarming with hostile natives, and a march of 150 miles under the circumstances presents many difficulties.

### The End of the American Coal Strike.

The coal strike in America came to an end last week, having lasted 23 weeks, and having cost the United States, it is said, about 150 million dollars. The coal owners and the miners have both consented to a commission of six being appointed to inquire into the questions at issue, the men in the meantime agreeing to return to work. This settlement is looked upon as the direct result of Mr. Roosevelt's efforts, and even his bitterest opponents do not, in their delight at escape from an imminent public danger, grudge the President praise and congratulation. He acted against the advice of some of his most cautious counsellors, but came forward simply as the first citizen on behalf of all citizens. The commissioners will make their report to President Roosevelt, who will communicate their findings to the parties interested and to the public. Work was resumed on Thursday.

### Pictures in Pall Mall.

The Royal Society of British Artists opened its 113th Exhibition to the public on Monday in Suffolk Street, Pall Mall. Mr. Watts, notwithstanding his eighty years, contributed a portrait of no mean merit. It is entitled "Miss Lilian Mackintosh," and depicts a young girl with pale golden hair, a white dress, a red sash, and a yellow scarf. The varied hues are not inharmonious, and the vigour displayed in the workmanship would do credit to many a younger man. Several of the most striking canvases are by ladies. Mrs. Lea Merritt has chosen the title of "The Helping Hand" for a religious allegory. In a bright-coloured cornfield a hard-featured peasant mother sits asleep with a child on her lap, and close by is the father apparently worn out by his toil. Christ, in shining raiment, stands beside the pair, and reaches out a helping and a cheering hand to the man. The figure of Our Lord is somewhat too conventional, but the face has dignity, and the roughness of the peasant group is well portrayed. Mrs. Jopling contributes a capital portrait of Mrs. Kendal in "The Merry Wives of Windsor." There are also portraits of "Miss Nellie Farren," by Mr. Graham Robertson, and of "La Belle Otero," by Mr. Rupert Bunny. Two portraits by Mr. W. B. Thompson deserve mention, one of the artist himself as he sits smoking a pipe, in an easy attitude, being especially promising. Miss Kemp Welch is at her best in "The Forest Stream," where she depicts a group of horses in the cool shade of a wood, drinking at a pool. The horses, of course, are excellently drawn, and the landscape is a careful study. Mr. Cayley Robinson contributes "Twilight," which, though interesting, is somewhat unequal in treatment. A last word of praise must be reserved for Mr. Sheard's "Cornfield." The golden corn, the glowing atmosphere, the healthy looking man with his wife and children form a pleasant pastoral, painted evidently by an artist with a genuine love of nature and an observant eye.

### Notes and Queries.

**The Editor is always willing to answer in this column, without any fee, all reasonable questions, as soon as possible.**

**But the following rules must be carefully observed:—**

**1. Every communication must be accompanied by the name and address of the writer.**

**2. The question must always bear upon nursing, directly or indirectly.**

**If an answer is required by letter a fee of half-a-crown must be enclosed with the note containing the inquiry, and we cannot undertake to forward letters addressed to correspondents making inquiries. It is therefore requested that our readers will not inclose either a stamp or a stamped envelope.**

*Abroad.*

(19) Will you kindly tell me to whom I should apply in order to join a nursing institute, and also where I could obtain private nursing on the Continent?—*Daisy.*

You might apply to the Nice Nursing Institute, Villa Pilate, Avenue Desambrois, Nice, or the Anglo-American Nursing Home, 265 Via Nomentana, Rome.

I am a fully-trained nurse and would like to obtain private work in South Africa; will you kindly let me know to which town it would be best for me to go?—*K. W.*

It is quite impossible to say. Go where you have an introduction.

Will you kindly give me the names of nursing homes in Rome and Switzerland where English nurses are employed?—*J. G.*

See reply to *Daisy*. The Hon. Secretary (address, 33 Curzon Street, W.), The Davos Invalids' Home, Davos, Switzerland, would be able to give you information as to Switzerland.

I have been nursing in the South of France in connection with an English institute, and should like to join a similar institution in Rome.—*Nurse Naomi.*

See reply to *Daisy.*

Will you kindly give me the name of any nursing home in Rome where an English lady could obtain an English nurse?—*A. B. S.*

See reply to *Daisy.*

*Hospital Training.*

(20) I wish to be trained as a nurse in typhoid cases. Where can I obtain typhoid germs in order to study them under a microscope?—*G. B.*

You might apply to the Birmingham Urban District Hospital for Infectious Diseases, Lodge Road, Birmingham. The instrument makers will obtain bacteriological slides for you.

Can you kindly tell me if there is any large London hospital which would give me two years' additional training with small salary? I have been some years in private work with a one year's certificate.—*S. M. K.*

No large hospital will do this, and it is by far the best plan to enter a training school for a three-years' certificate. Excellent training is given, with salary from the first. The extra year's service required is amply compensated by the preference accorded to those holding a certificate for three years' continuous training against the possessors of certificates acquired at more than one school.

Will you kindly tell me if there is a children's hospital in Yorkshire where I could get a good training? Please say if being stout, through perfect health, would disqualify me?—*M. N.*

Apply for information to the Matron, the Bradford Children's Hospital, Bradford. No, certainly not.

Will you kindly tell me if the three-years' certificate from the Central London renders the possessor eligible to enter any hospital as well as infirmaries as sister?—*Truth.*

There is no reason against it. Candidates for appointments are chosen for various reasons, and a three-years' certificate in general nursing is one of the indispensable qualifications in most cases. Certificates from Poor Law institutions, although qualifying a nurse fully, do not always carry the same weight for important posts as those from the London hospitals which have established a high reputation for the perfection of their nursing departments.

*Symptoms.*

(21) I should like to know the best thing to do when menstruation ceases in a young person for over three months. Is it injurious to health, and would it be the cause of a skin-eruption on the face and neck?—*L. S.*

It is probable that the same cause lies at the root of the skin-affection and of the symptoms you mention. You must put the case under the care of a reliable physician.

*Books.*

(22) I should esteem it a favour if you could kindly tell me the name of a book published by the Medico-Psychological Association for mental attendants.—*Nurse K.*

Write to the Secretary of the Medico-Psychological Society, 11 Chandos Street, W.

I want to get a medical dictionary larger than Miss Honnor Morton's, and also a small book on urine-testing.—*Elizabeth.*

There are several large medical dictionaries published, as well as smaller pocket editions. If you write to the Manager of the Scientific Press, he is always ready to give information to nurses on the books relating to their calling and to procure them. Probably the dictionary best suited to your use would be Holbyn's "Urine Testing," by Dr. Robinson, price 1s., obtainable from the Manager of the Scientific Press.

*Mental Nursing.*

(23) Can you kindly tell me the best place at which to get good mental training, in or quite near a town? I have had three years' experience in a private home where slight mental cases were taken. How long should I have to train?—*M. T.*

You should if possible get employment in one of the asylums where the attendants receive three years' training for the certificate of the Medico-Psychological Society. The Northampton County Asylum, Berry Wood, near Northampton, is one of the very best.

I am a nurse who has had asylum experience, but I have not the certificate of the Medico-Psychological Association. Will you tell me if I can prepare for it whilst engaged in private work? Please give me particulars.—*E. C.*

You must be in an asylum, and be trained in general nursing in the infirmary wards as well, to be eligible for this examination. One year's training in the infirmary would probably be sufficient in your case. You will, however, obtain all particulars by applying to the Registrar, 11 Chandos Street, W.

*Paper.*

(24) Can you tell me if there is a paper in South Africa similar to The Hospital, with advertisements, etc., and also how I could obtain it?—*P. K.*

There is none, but you could obtain information as to the best means of advertising in the Colony at the Emigration Information Office, the Broadway, Westminster, S.W.

*Morphia.*

(25) Will you kindly tell me if there is any institution in England where, for moderate fees, a cure is guaranteed for the morphia habit?—*M. R. D.*

No cure can be guaranteed for the morphia habit. The utmost that can be done for its victims is to keep them for a long period under strict supervision with, of course, suitable medical care. Advertise for exactly what you want.

*Instep Supports.*

(26) I should be very glad indeed if you could tell me of a good instep supporter. I have a pair made by a surgical-instrument maker, but the weight is an added discomfort. Can you tell me of a maker?—*Nurse Dorothy.*

Messrs. Garrould, Edgware Road, W., Messrs. Goochs, Limited, 67 and 77 Brompton Road, S.W., and Messrs. Pond, 21A Castle Meadow, Norwich, all supply these articles. Messrs. Gooch make the support in their ward shoes; whilst Messrs. Ponds' are available for any shoe, and do not take up extra room.

*Feeding-bottle.*

(27) I should like to know the name and address of the manufacturers of the boat-shaped feeding-bottle, stamped "The Foster Mother."—*J. G.*

You local chemist will get it for you.

*Lady Dispenser.*

(28) Can anyone give me full information how to become a lady dispenser?—*Nurse R.*

Apply the Secretary of the Pharmaceutical Society, 17 Bloomsbury Square, W.C.

**Standard Books of Reference.**

**"The Nursing Profession: How and Where to Train." 2s. net; post free 2s. 4d.**

**"Burdett's Official Nursing Directory." 3s. net; post free, 3s. 4d.**

**"Burdett's Hospitals and Charities." 5s.**

**"Hospital Expenditure: The Commissariat." 2s. 6d.**

"The Nurses' Dictionary of Medical Terms." Cloth, 2s.; leather, 2s. 6d. net.; post free, 2s. 8d.

**"Burdett's Series of Nursing Text-Books." 1s. each.**

**"A Handbook for Nurses." (Illustrated). 5s.**

**"The Physiological Feeding of Infants." 1s.**

**"The Physiological Nursery Chart." 1s.; post free, 1s. 3d.**

**All these are published by the Scientific Press, Ltd., and may be obtained through any bookseller or direct from the publishers, 28 and 29 Southampton Street, London, W.C.**

### Travel Notes.

**By Our Travelling Correspondent.**

### CXII.—A ONE DAY'S EXCURSION.—ST. ALBANS.

This has not been a good year for those pleasant little trips comprised within a one day's holiday, which make such an agreeable break in nursing work, when we gather fresh energy with the clear air and bright restful green of the fields surrounding London; but we still have fairly warm autumn days, though night falls sadly early now. It was about this time last year that I went with an energetic friend for a day's rest at St. Albans. We were to take a combined meal of lunch and tea fitted into a capacious Pyrenean basket, and my intention was to be strictly idle. Alas! for ill-founded hopes. On meeting in the early morning at my flat I was sternly questioned as to what preparations I had made for sketching. Guiltily conscious of having made none at all, I meekly suggested that it was a little cold for prolonged sitting out of doors, but my feeble subterfuges were swept away. "You can be putting your things together whilst I re-arrange the tea-basket!" I did as I was told, and eventually succeeded, under the watchful eye of the energetic one, to secure a very pretty distant view of the Abbey.

### Cost of the Journey.

This comes to 3s. 3d. return third-class, and with a well-stocked basket it is quite unnecessary to go to an inn or confectioner's. From Euston you reach the old town in three-quarters of an hour. From the station to the Abbey is rather a long walk, and it is uphill, but the return seems nothing.

### The Abbey.

One must at once admit that the building is so largely restored that a very considerable part of it is really rebuilt, but it has been very judiciously done by the generous gifts of Lord Grimthorpe. As a rule restorations are an abomination to me, but the church was in such a neglected condition, and was so rapidly falling into decay, that, had its renovation not have been attempted, it would have soon passed beyond the possibility of artistic repair. On the whole, it has been very well done, though in places the restorations have been made with sadly little attention to the original structure. Still, anything is better than the neglect of past years. Behind the Lady Chapel it is clearly visible where but a short time since a public path cut right through the ruins!

A charge of sixpence is made to enter the sanctuary, but once inside you are left to yourself, and not worried by vergers. The most interesting thing in the whole church is—at least to me—the shrine of St. Alban, and the watching loft which overlooks it.

Of the shrine itself hardly anything remains, and the pedestal on which it stood, was ruined and scattered in all directions; the fragments have now been collected and most beautifully and carefully pieced together. There are two apertures piercing the pedestal, which the learned are of opinion were used for the insertion of diseased limbs, whilst the sufferers prayed for the saint's aid. Of the shrine proper, from an account in the Cottonian MSS., it appears to have been of the shape of a church, and had a silver-gilt tower.

Whilst repairing the pavement near this spot a very quaint seal was found, which gives one a good idea of the military equipment of a soldier in those days. The designer evidently experienced great difficulty in representing the warrior's eye in profile, with the singular effect that the figure is purely side faced, whilst the eye glares at you as if in front view.

Close to the shrine is the lovely watching loft, which is, with the exception of one in Oxford (Christ Church), the only existing perfect specimen in England—at least so I am told.

The style is Perpendicular, of the early fifteenth century; it bears the badge of Richard II., the white hart, and this would seem to point to a somewhat earlier date. At the east end is a narrow stair by which the watching monk ascended to his post. The carvings are most interesting and quaint, though much injured. The months are very curiously represented—January and February by peasants sitting at a fire feasting and using bellows; March by a shepherd and sheep; April by a lamb; May by a woman milking; and so on. In front of the high altar Queen Eleanor's body rested as her husband was bringing it to Westminster. There was an Eleanor Cross in the High Street which was removed in 1700, and what became of it I could not find out.

### The Town.

I saw it on a brilliant October day last year, and as the market was in full swing, the effect was quite gay and foreign-looking. There are still many curious overhanging houses and quaint nooks, and it would be a good sketching-ground. About three-quarters of a mile from the town are the remains (mere indications) of the nunnery, and for this point we made, the energetic one assuring me that I should have a good distant view of the Abbey, which was quite true. At a picturesque old cottage which I made the foreground of my picture, we got the good woman to make our tea for us, and we eat, drank, and rested very comfortably in the meadow in front of her domain, returning as the darkness began to fall.

For cyclists it is a nice and really easy run, and not more than 20 miles each way. The country round is very pretty in a mild way, and would be worth exploring. Passing the Cathedral, there is a pretty road or rather lane that would make a good picnic ground, and there I wished to penetrate for tea, but the energetic one would not hear of it till the sketch was finished, and by that time alas! the short October daylight was waning. I hope from time to time to give accounts of interesting places to be seen in one day from London. It would, I think, often be more beneficial to nurses to spend their monthly day more in the open air, and what can be nicer than with a chosen friend to visit some such interesting spot as St. Albans, speaking so plainly of a glorious past.

### TRAVEL NOTES AND QUERIES.

Winter in Pau (Hedwig).—If you will send me a stamped and addressed envelope I will give you the address you want. It is private and not for publication. I cannot entirely recall your former letter.

### Wants and Workers.

Will any kindly-disposed lady having any disused warm clothing allow district nurse Haverland, Cawston, Norfolk, to have such for free distribution in a very large poor district? Anything suitable, either for old women or infants, will be gratefully received and thankfully acknowledged.

